# Sustainable Synthesis of Highly Biocompatible 2D Boron Nitride Nanosheets

**DOI:** 10.3390/biomedicines10123238

**Published:** 2022-12-13

**Authors:** Marina Llenas, Lorenzo Cuenca, Carla Santos, Igor Bdikin, Gil Gonçalves, Gerard Tobías-Rossell

**Affiliations:** 1 Institut de Ciència de Materials de Barcelona (ICMAB-CSIC), Campus de la UAB, 08193 Bellaterra, Spain; 2 LAQV-REQUIMTE, Department of Chemistry, University of Aveiro, 3810-193 Aveiro, Portugal; 3 CQE—Centro de Química Estrutural, Instituto Superior Técnico, University of Lisbon, 1049-001 Lisbon, Portugal; 4 TEMA-Nanotechnology Research Group, Mechanical Engineering Department, University of Aveiro, 3810-193 Aveiro, Portugal; 5 Intelligent Systems Associate Laboratory (LASI), 3810-193 Aveiro, Portugal

**Keywords:** sonication, biocompatibility, green chemistry, 2D materials, layered compounds, cytotoxicity, surfactant, pluronic acid F127, functionalization, BNCT

## Abstract

2D ultrafine nanomaterials today represent an emerging class of materials with very promising properties for a wide variety of applications. Biomedical fields have experienced important new achievements with technological breakthroughs obtained from 2D materials with singular properties. Boron nitride nanosheets are a novel 2D layered material comprised of a hexagonal boron nitride network (BN) with interesting intrinsic properties, including resistance to oxidation, extreme mechanical hardness, good thermal conductivity, photoluminescence, and chemical inertness. Here, we investigated different methodologies for the exfoliation of BN nanosheets (BNNs), using ball milling and ultrasound processing, the latter using both an ultrasound bath and tip sonication. The best results are obtained using tip sonication, which leads to the formation of few-layered nanosheets with a narrow size distribution. Importantly, it was observed that with the addition of pluronic acid F127 to the medium, there was a significant improvement in the BN nanosheets (BNNs) production yield. Moreover, the resultant BNNs present improved stability in an aqueous solution. Cytotoxicity studies performed with HeLa cells showed the importance of taking into account the possible interferences of the nanomaterial with the selected assay. The prepared BNNs coated with pluronic presented improved cytotoxicity at concentrations up to 200 μg mL^−1^ with more than 90% viability after 24 h of incubation. Confocal microscopy also showed high cell internalization of the nanomaterials and their preferential biodistribution in the cell cytoplasm.

## 1. Introduction

A wide range of methodologies has been reported in the literature to obtain single layers of van der Waals solids that have unique properties compared to their bulk counterparts [1,2,3,4,5,6,7,8]. Boron nitride nanosheets (BNNs) have been produced by several different approaches, including micromechanical, unzipping BN nanotubes, chemical vapor deposition (CVD), and solution-processed exfoliation. BNNs’ sonication-assisted exfoliation has been one of the most explored approaches for the attainment of high-quality and large-scale production. For improving the efficiency of the exfoliation process, the surface tension, molecular weight, and chemical structure of the solvents are critical in order to match the surface tension (≈35 mJ m^−2^) and consequently minimize the exfoliation energy. Coleman et al. developed a systematic study to determine the Hansen solubility parameters of BN according to the production yield of BNNs in a range of solvents. Their findings determined that isopropyl alcohol (IPA) is one of the best solvents for the preparation of BNNs (0.06 mg mL^−1^) [9]. The high solvent polarity of ionic liquids was also investigated in order to increase the exfoliation yield BNNs while retaining lateral size. Morishita et al. reported a highly efficient exfoliation of h-BNs by sonication using [bmim][PF6] and obtained a suspension of BNNs with a concentration of ∼1.9 mg mL^−1^ [10]. Marsh et al. evaluated the h-BN exfoliation yield by using a range of co-solvent systems at different ratios of water/organic solvents, including acetone, methanol, ethanol, 2-propanol, 1-propanol and tert-butanol [11].

Lei et al. reported the ball milling exfoliation of h-BN by using urea [12]. The obtained few-layer BNNs demonstrated an impressive water solubility of up to 30 mg mL^−1^. The authors attribute this high solubility in water to the high surface functionalization of BNNs with amino functional groups during the synthesis process. Recently, a multistep method was reported, starting with thermal treatment at 900 °C for h-BN, then air plasma treatment, and finally sonication with different solvents. They reported the formation of OH-functionalized BNNs with a high yield of ca. 80% and a large concentration of 5 mg mL^−1^ when DMF was used in the sonication medium [13]. However, the use of non-environmentally friendly solvents, as well as the need for additional purification processes, make the co-solvent synthesis of BNNs unappealing, especially when BNNs were investigated for biomedical applications [14,15].

Several additives have been explored to assist the exfoliation of h-BN in water, including Lewis base molecules [16], methanesulfonic acid (MSA) [17], sodium cholic acid (SC) or sodium deoxycholic acid (SDC) [18], and a variety of inorganic reagents, including hydrazine, H_2_O_2_, HNO_3_/H_2_SO_4_ [19], among many others [20].

Surfactants have also been widely explored for water exfoliation of h-BN. Zhu et al. describe the tip sonication synthesis of BNNs using Pluronic F68 as a dispersant. The purified BNNs were obtained by isopycnic density gradient ultracentrifugation [21]. Smith McWilliams et al. reported a comparative study about the effectiveness of h-BN exfoliation by sonication in an aqueous solution, using different surfactants (1 wt. %). According to their findings, at low centrifugal forces, large-molecular-weight nonionic surfactants allowed for highly concentrated dispersions, while at higher centrifugal forces, dispersion ionic surfactants contained significantly higher concentrations of BNNs with improved stability [22]. Indeed, they found that cationic surfactants, especially DTAB, were the best for improving the exfoliation and dispersion of BNNs. Recently, a two-step method was reported, where the pristine h-BN powder was firstly thermally treated at 500 °C and then processed by ball milling in the presence of glucose as a dispersant agent [23]. For the best experimental conditions, the obtained and exfoliation yield of BNNs was 47%, with a high aspect ratio and few lattice defects that mostly correspond to the formation of functional hydroxyl groups. A new approach for the production of BNNs has been based on h-BN freezing-induced volumetric expansion under liquid nitrogen and subsequently ultrasonically (300 W). The optimized experimental conditions showed that the combination of five freezing and exfoliation cycles allowed for the formation of single-layer BNNs. The authors reported a maximum exfoliation yield of thin BNNs (<10 nm) of 58%, with their surface being functionalized with hydroxyl groups [24].

The use of nanosheets, and nanoparticles, in general, in the biomedical field offer a unique platform to adjust essential properties such as solubility, diffusivity, blood-circulation time, pharmacokinetic profile and cytotoxicity. Therefore, several nanoformulations are being explored in a wide variety of biomedical applications [25,26,27,28,29,30,31,32]. Self-assembled boron nitride nanosheets have been recently applied to boron neutron capture therapy (BNCT), being useful for breast cancer treatment and showing their potential in biomedicine applications [33]. Despite the interesting application of BNNs in the biomedical field, only a few studies have been conducted to understand their toxicity and some of them present conflicting results [15,34]. For example, sheet-like BN materials were reported to present toxicity and cause adverse effects on human hepatoma HepG2 cells, such as reducing cell viability, increasing ROS generation, or inducing damage to membrane integrity [35]. Czarniewska et al. [36] showed that BNNs functionalized with hydroxyl groups (h-BN-OH-n) presented low-cytotoxicity on insect hemocytes (in vivo), human erythrocytes and mouse L929 cells (in vitro). However, despite having low cytotoxicity, a long-term study in the *Tenebrio molitor* beetle showed that h-BN-OH-n can significantly affect immunocompetent cell behavior, which in turn affects their function during the immune response; it is necessary to study the in vivo long term effects in different animal models. The in vivo toxic effects of BNNs have also been evaluated in silkworm models continuously feeding with different mass concentrations (1%, 2%, 3%, 4%), and evaluating animal entirety, tissues and genes, showing that the exposure to BNNs do not cause obvious adverse effects but the expressions of genes in the midgut concerned with some specific functions and pathways are significantly changed, indicating that BNNs may have the potential danger to lead to dysfunction [37]. The hemolytic effect has also been studied showing that BNNs cause damage to red blood cell membranes [38].

Mateti et al. [14] reported that the size, shape, structure and surface chemistry directly affect BNNs biocompatibility in a dependent manner, showing that lower sizes were less biocompatible on human osteosarcoma cells. Coatings have been shown to present a high effect on the nanomaterials’ biocompatibility [15], being pluronic, a non-toxic surfactant can significantly improve the biocompatibility and reduce the cytotoxicity of the coated nanomaterials [15,39,40].

In the present study, the cytotoxicity of the developed BNNs by bath and tip sonication that present a pluronic F127 coating is going to be evaluated. Pluronic F127 was employed in the present study because we have previously employed this surfactant to increase dispersibility and in turn, the biocompatibility of low-dimensional materials [40,41,42]. Some studies have shown that layered materials can present some interferences with common cytotoxicity assays inducing errors in results interpretation. This demonstrates the importance of carefully interpreting the obtained data from in vitro cytotoxicity assays [43,44]. Different methods will thus be performed to elucidate possible interferences on the registered data in vitro. For their future biomedical application, it is important that the nanomaterials efficiently internalize into targeted cells to improve the therapeutic effects. For example, for boron neutron capture therapy a critical 10-boron cell uptake threshold should be enriched to obtain therapeutic effects [45], and several materials are being developed for this purpose [46,47,48,49]. Importantly, BNNs internalization is highly affected by their final properties including size, shape, or surface properties such as charge [50,51]. For this reason, cellular uptake of the synthesized BNNs is an important aspect to be evaluated in order to successfully reach pre-clinical studies.

## 2. Materials and Methods

### 2.1. Materials

h-BN powder (Merck KGaA, Darmstadt, Germany), Isopropanol (Merck KGaA, Darmstadt, Germany), Benzyl benzoate (Merck KGaA, Darmstadt, Germany), pluronic acid F127 (Merck KGaA, Darmstadt, Germany).

### 2.2. Exfoliation of h-BN by Sonication

The exfoliation of h-BN (1.25 g) was carried out by dispersing the as-received material in 45 mL of distilled water, with and without the addition of pluronic acid F127(3.15 g) as a surfactant. The exfoliation of BNNs was first performed by using the tip sonication Q700 Sonicator Qsonica (Qsonica, Newtown, CT, USA), with controlled applied power at 80 W and 200 W. A comparative study was also implemented by using bath sonication (Bransonic^®^ CPXH 3800 Branson, BRANSON Ultrasonidos, L’Hospitalet de Llobregat, Barcelona, Spain). The temperature of the bath sonication was fixed at 50 °C. The time for the exfoliation of h-BN was 8 h and 24 h. Finally, all the obtained samples were purified twice by centrifugation at 4500 rpm for 20 min. The deposited material corresponding to the BNNs with larger thicknesses was discarded and the supernatant with thinner BNNs was collected and centrifuged again using the same conditions. Again, the supernatant was collected and ultracentrifuged at 20,000 rpm for 20 min. In this case, the supernatant was removed and the deposited materials were redispersed in water and centrifuged again to remove the pluronic excess. The resulting deposit was dispersed in distilled water and kept in the fridge for future characterization. To determine the concentration of the BNNs solutions, an Eppendorf was weighed and then a known volume was added to it. The solution was dried and the Eppendorf was weighed again, knowing that the difference corresponds to the sample weight. Therefore, knowing the sample weight and the corresponding volume, the concentration could be calculated in each case.

### 2.3. Exfoliation of h-BN by Ball Milling

The synthesis of BNNs by ball milling was performed by changing several experimental parameters, including solvents (isopropanol and benzyl benzoate), time (8 and 24 h) and ball diameters (1.0 and 5.0 mm). Initially, 0.5 g of h-BN (Sigma-Aldrich, St. Louis, MO, USA) was mixed with 47.5 mL of the chosen solvent and inserted into the milling jar. After that, the exfoliation was conducted using a planetary ball miller (PM100, Retsch, Haan, Germany), by adjusting the time at a constant speed of 400 rpm. The obtained BNNs were purified by centrifugation initially at 1500 rpm and then at 4500 rpm.

### 2.4. Characterization

Exfoliated BN were analyzed by attenuated total reflectance Fourier transform infrared (ATR-FTIR) in a Bruker Tensor 27 FT-IR spectrometer (BrukerCorporation, Billerica, MA, USA). Spectra were recorded between 400 and 4000 cm^−1^.

UV-Vis spectroscopy (Shimadzu UV-Vis 1700, Shimadzu Corporation, Analytical Instruments Divison, Kyoto, Japan) was performed from 200 nm to 1200 nm measuring the absorbance of the BNNs dispersed in water at different concentrations (2, 20, 50, 100 and 200 µg mL^−1^). Water was used to make the background and h-BN powder was also analyzed. A Fluorolog-3 (Horiba, Kioto, Japan) spectrofluorometer was used to collect the emission spectra. The fluorescence quantum yield was determined relative to a fluorescence standard. The luminescence quantum yield was obtained by integrating the emission in the 320–600 nm range upon excitation at 300 nm and using a cresyl violet perchlorate solution in methanol (ϕ = 0.54) as a reference.

Thermogravimetric analysis (TGA, SDT 650, TA instruments, New Castle, DE, USA) was performed under argon flow, from room temperature up to 873.15 K at a heating rate of 10 K min^−1^. Morphological features of h-BN were analyzed using scanning electron microscopy (SEM) Quanta FEI 650FEG. The transmission electron microscopy (TEM) and high-resolution TEM (HRTEM) images were acquired with HRTEM Tecnal F20 and TEM JEOL 1210, respectively. Atomic force microscopy (AFM) images were recorded on an SPM Nanoscope III multimode microscope equipped with an RTESPSS tip (Veeco, Plainview, NY, USA).

Elemental analysis (Flash EA 2000 CHNS, Thermo Fisher Scientific, Waltham, MA, USA) was performed to determine the presence of P-F127. CNHS was determined by combustion at 1200 °C in an oxygen atmosphere and quantified by gas chromatography. Two replicas were performed for each sample and the mean value was calculated. The detection limit of the equipment is 0.1%.

### 2.5. In Vitro Studies

#### 2.5.1. Cell Culture

HeLa cells, coming from a human cervix epitheloid carcinoma, were cultured in MEM alpha w/glutamax-I supplemented with 10% fetal bovine serum at 37 °C in a humidified atmosphere of 5% CO_2_ in air. Cells were trypsinized when they arrived at 80% confluence.

#### 2.5.2. In Vitro Cytotoxicity

To evaluate the cytotoxicity of the synthesized BNNs a modified MTT assay (EZ4U, Biomedica, Vienna, Austria) was employed. This assay quantifies mitochondrial activity and thereby cell viability, as viable cells have an active metabolism and are able to reduce tetrazolium salts to formazan (colored, optical density at 450 nm).

To perform the study, HeLa cells were seeded on a 96-well plate using 100 µL of cell suspension having 3.5 × 10^3^ cells/well. BNNs’ dispersions were sterilized with UV light for 1 h and different concentrations were prepared (4, 40, 100, 200 and 400 µg mL^−1^). Cells were allowed to grow for 24 h and then 100 µL of a dispersion of nanosheets at different concentrations were added, having a final volume of 200 µL in each well and consequently half of the initially prepared concentrations (2, 20, 50, 100 and 200 µg mL^−1^). Once BNNs had been added, cells were incubated for an additional 24 h. Controls were prepared by adding 100 µL of culture medium to the wells with only cells and 100 µL of culture medium or BNNs solution to the wells without incubated cells. Finally, 20 µL of tetrazolium salts solution (EZ4U kit) was added to each well in order to determine the viability of the cells. Optical density was measured in a Multilabel Plate Reader VICTOR3 after 3 h of incubation. Two different wavelengths were employed, 450 nm to measure the absorbance of formazan, and 620 nm, as a reference. Knowing the absorbance value of the wells with cells and BNNs, and the values of the different controls, the cell viability in each case could be determined, considering that the mean value of the wells with cells without BNNs (untreated cells control), subtracting the contribution of the medium, was fixed at 100% of viability and used as reference.

These nanomaterials can interfere with the aforementioned colorimetric assay, altering the viability results and, accordingly, falsifying results. To avoid their possible interferences, an additional study was performed; 96-well plates were prepared in the same manner, but before adding the EZ4U reagent, all the wells were emptied, cleaned with PBS and then filled with the new medium. After that, 20 µL of tetrazolium salts were added.

Finally, to further analyze the cytotoxicity of the BNNs, flow cytometry was performed; 6-well plates were seeded with 3 × 10^4^ cells/well and incubated for 24 h. Then, BBNs were added using the same concentrations and left in contact for 24 h. After the incubation time, each well was trypsinized, keeping the medium of each well as death cells were suspended on it. Then they were centrifuged and resuspended in PBS to be analyzed with a flow cytometer (FACSCanto, BD Biosciences). Propidium iodide (2 µg mL^−1^) was used to stain dead cells. Different controls were used to correctly determine the cell death population and avoid BNN interference [52].

#### 2.5.3. Cell Internalization

BNNs’ internalization was evaluated in HeLa cells using confocal microscopy through their direct detection by light reflection. Sterile 24 mm × 24 mm coverslips were used to prepare the sample, they were put inside a 35 mm dish and 3 × 10^4^ cells were seeded on them. Sterile nanosheet solutions were mixed with medium to obtain two different concentrations of 100 µg mL^−1^ and 400 µg mL^−1^. After 24 h of incubation at 37 °C and 5% of CO_2_, 1 mL of BNNs solution was added to each dish without removing the previous milliliter of medium obtaining a final concentration of 50 µg mL^−1^ and 200 µg mL^−1^. Cells were left to grow for 24 h in contact with the nanomaterial, then the medium was removed and cells were washed with PBS. After that, cells were fixed with paraformaldehyde (PFA) 4% for 20 min and washed with PBS. HeLa cells were dyed with Cell Mask Deep Red Membrane 5 mg mL^−1^ (Invitrogen^TM^, Waltham, MA, USA), which stains the plasma membrane and with Hoechst 33342 10 mg mL^−1^ (Invitrogen^TM^, Waltham, MA, USA), which stains the nuclear DNA; 1 mL of dye solution (1:1000) was added to each dish and cells were incubated for 10 min under room temperature and protected from light, followed by a washing step with PBS, staining first the membrane and then the nucleus. Finally, coverslips were transferred to a glass slide using mounting media prolong glass to attach them.

For image acquisition, Leica TCS SP5 (Servei de Microscòpia de la UAB, Cerdanyola del Vallès, Spain) was employed. The excitation laser lines used for each dye were 633 nm (5%) for Cell Mask Deep Red Membrane, 405 nm (40%) for Hoechst 33342 and 488 nm (10%) for BNNs reflection. The obtained images were processed using ImageJ software (version 1.8.0).

## 3. Results and Discussion

### 3.1. Synthesis and Characterization of BNNs

**Figure 1:**
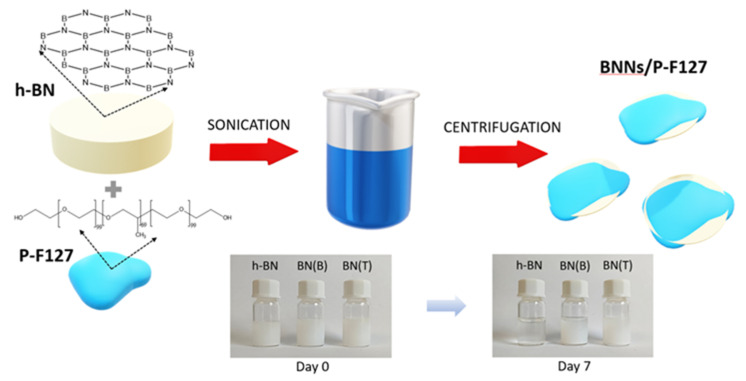
Schematic representation of the synthesis of BNNs by ultrasound-based approaches in water solution and P-F127. The photographs show the stability of the samples over 7 days in water. As it can be seen, despite as-received h-BN precipitates, a good stability is observed for the P-F127 coated BNNs.

We explored different scalable strategies for the exfoliation of biocompatible BNNs based on sonication and ball milling. A systematic study was performed for the synthesis of BNNs using both tip and bath sonication (Figure 1). The role of the exfoliation time, in the case of bath sonication, and the applied power, in the case of tip sonication, were investigated. Our results showed that exfoliation of h-BN in water solution by the tip is strongly dependent on the increase in power from 80 W to 200 W, which results in a significant increment of the BNNs’ yield from 11.3 wt. % up to 30.7 wt. % (Table 1). In the case of bath sonication, an exfoliation yield of 11.2 wt. % was obtained after 8 h and 25.7 wt. % after 24 h treatment at 50 °C. These results clearly indicate the high efficiency of the exfoliation of BNNs by the tip sonication method in terms of mass ratio. Next, we also investigated the effect of the addition of a surfactant, pluronic acid F127 (P-F127), on the yield of exfoliation and the water stability of the BNNs. With the addition of the dispersant agent P-F127 to the h-BN water solution, we observed a significant increment in the exfoliation yield of BNNs by tip sonication (80 W) for 28 wt. %. This exfoliation yield of BNNs is similar to the one observed for tip sonication of h-BN in a pure water solution with a higher power (200 W). The use of 200 W of power in a BN solution with the surfactant P-F127 resulted in a large degradation of the starting materials. In the case of bath sonication, the addition of P-F127 also allowed reducing the exfoliation time. A similar production yield was obtained at 8 h in the presence of P-F127 than at 24 h with ultrapure water. However, is important to notice that when a 24 h reaction time with P-F127 was used, a large degradation of BN was observed.

Ball milling was also explored in this work for the exfoliation of h-BN. Since the traditional ball milling technique (without solvents) normally causes considerable damage and surface defects, liquid agents were chosen to reduce this impact. Several experimental parameters were investigated in order to maximize the exfoliation yield, including the type of solvent, time, and diameter of the steel balls. In the first attempt, IPA was used as a solvent, since it is relatively low-priced and easy to purify from the resultant BNNs (Table 2). The best results were obtained by exfoliating the BN material for 8 h, with 5 mm diameter steel balls (Figure S1). Maintaining the same exfoliation conditions but using steel balls with a diameter of 1 mm (keeping the weight ratio of the material-weight of the balls constant), fragmentation of BN particles was preferentially obtained to the detriment of the exfoliation process. This effect can be attributed to the higher number of balls used with smaller diameters, which causes more collisions of the steel balls with the h-BN during milling, favoring in this way the fragmentation effects. Maintaining the exfoliation for 24 h, with steel balls with a diameter of 5 mm, a partial fragmentation, and a small reduction in the size of the material were observed (Figure S1). Benzyl benzoate (BB) was chosen as an alternative to IPA given the good performance on the exfoliation of BNNs reported in the literature [53,54]. This behavior was attributed to BB’s high density and surface energy, which is similar to that of h-BN, favoring in this way its exfoliation. For a comparative study with BB, balls 5 mm in diameter were employed since they gave the best exfoliation with the IPA adopted (Table 2). After 8 h treatment, a low degree of exfoliation of h-BN was observed, having predominantly a reduction in the size of the particles, while maintaining a considerable thickness (Figure S2). This fact could be due to the high density and viscosity of BB compared to IPA, which considerably reduces the shear forces produced during milling. It was also difficult to recover the BNNs from the BB solution. After 24 h, a significant increase in the fragmentation of the h-BN was observed, making it extremely difficult to recover the particles from the BB solution.

Taking into account the analysis performed so far, exfoliation by ultrasound in the presence of P-F127 arises as the most promising strategy for the production of BNNs. Furthermore, the prepared samples with P-F127 present excellent water dispersibility, thus facilitating its further processing. Thus, the best conditions for the exfoliation of h-BN using ultrasound-based methodologies were chosen for further characterization. In the case of the tip sonication method, the exfoliation of h-BN in water solution was adopted with the addition of the surfactant P-F127 (BN(T)), due to the high exfoliation yield of 28 wt. % at lower power (80 W). For bath sonication, the exfoliation of h-BN in a water solution with the addition of P-F127 during 8 h was chosen, which allowed for obtaining a yield of 27.2 wt. % (BN(B)), similar to that reported in the case of tip sonication. Despite exfoliation without the addition of surfactant leading to a similar exfoliation yield, it requires higher energy consumption and is less environmentally friendly. Furthermore, the presence of P-F127 is of great interest for the potential application of the exfoliated BNNs in biomedicine.

**Figure 2:**
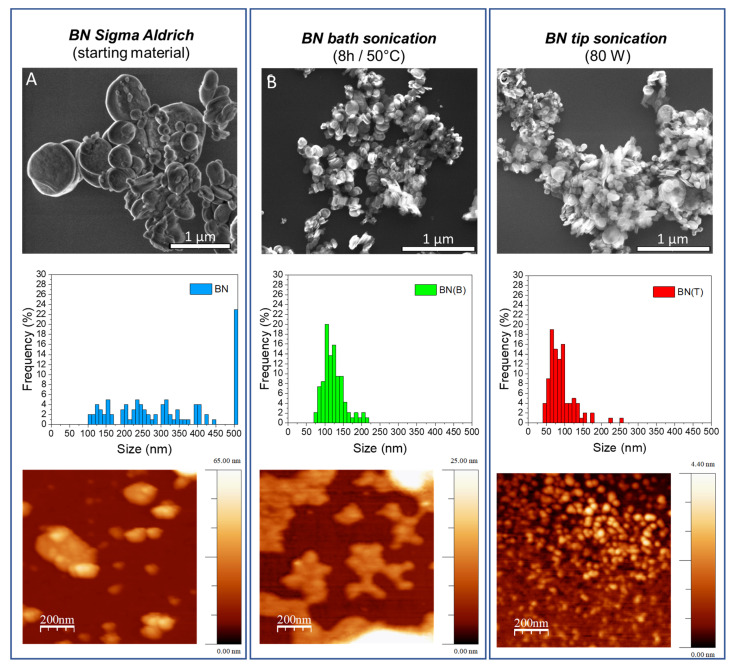
SEM images of (**A**) h-BN, and exfoliated BNNs in water with pluronic acid F127 by (**B**) bath or (**C**) tip sonication. Size distribution (**A**) h-BN, and (**B**,**C**) exfoliated BNNs determined by SEM analysis. Representative AFM images of (**A**) h-BN, and (**B**,**C**) exfoliated BNNs.

The structural analysis of the obtained BNNs (BN(T) and BN(B)) was performed in comparison with the starting raw material h-BN. The SEM analysis of the as-received h-BN showed a very broad size distribution of particles, with several of them having a diameter larger than 500 nm (Figure 2A). The BNNs exfoliated by bath sonication (BN(B)) showed an average diameter of 116.0 ± 1.2 nm (Figure 2B) and by tip sonication (BN(T)) showed an average diameter of 77.5 ± 1.4 nm (Figure 2C). Although SEM analysis did not allow us to obtain a conclusive analysis regarding the level of exfoliation of h-BN, these results clearly indicate that there is a stronger fragmentation of BN particles in the case of the tip sonication method. This can be understood by the higher energy delivery in the solution by this method, which is in agreement with our previously reported results on the high efficiency of tip sonication for the lateral size decrease in the graphene oxide nanosheets [55].

**Figure 3:**
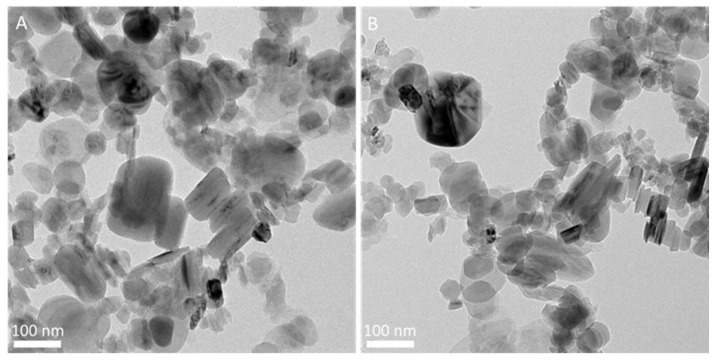
TEM analysis of the BNNs prepared by (**A**) bath or (**B**) tip sonication.

AFM analysis allows us to investigate the thickness and lateral dimensions of the pristine h-BN compared to BNNs exfoliated by tip or bath sonication. Our results show that the decrease in the dimensions of BNNs is related to the sonication treatment applied. Before ultrasonication treatment, the thickness of h-BN was approximately 24 nm (Figure 2A and Figure S3). The thickness of BN(B) was greatly reduced after ultrasonication treatment to ca. 6-8 nm (Figure 2 and Figure S3) observing slightly thinner sheets for BN(T) than for BN(B). The lateral dimensions determined by AFM are within the range determined by SEM analyses (histograms in Figure 2). TEM analysis also corroborates the results obtained by SEM and AFM analysis in terms of the different size distributions of BNNs prepared by bath or tip sonication. Figure 3A shows the presence of large exfoliated BNNs by bath sonication when compared with the ones prepared by tip sonication in Figure 3B. A sonication-assisted liquid-phase exfoliation method for the efficient synthesis of graphene and other 2D inorganic counterparts has been recently reported. They observed that at high frequencies, ultrasonic liquid phase exfoliation allowed a more effective weakening of the interlayer van der Waals forces holding the individual 2D layered sheets [56]. Additionally, it was also reported that the exfoliation of bulk-layered materials, including BN, and the dispersion of the exfoliated 2D nanosheets in pure water can be obtained by controlling the temperature of the sonication bath. The authors used heat dissipation from sonic energy to raise the temperature of the sonication bath [57]. Our results show that the highly energetic probe sonication method can improve ultrasonic cavitation for more effective exfoliation of the BNNs while also improving the fragmentation of the BNNs’ covalent bonds, which results in nanoparticles that are simultaneously thinner and smaller.

**Figure 4:**
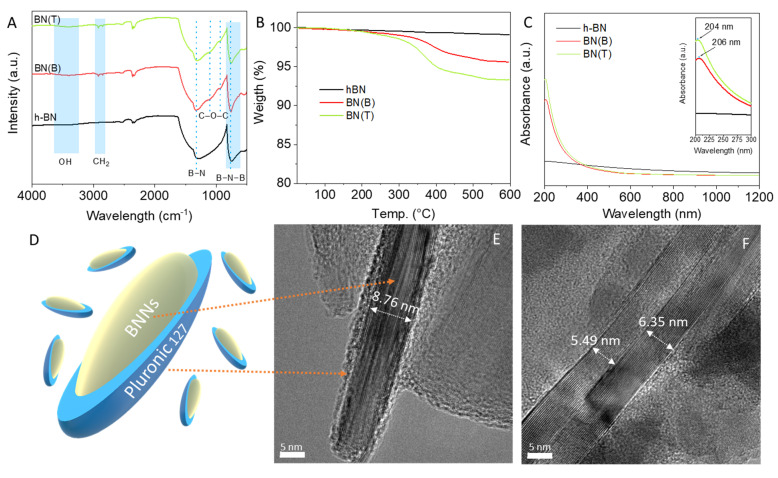
Characterization of synthesized BNNs by tip (BN(T)) or bath BN(B) sonication comparatively to the staring material h-BN. (**A**) FTIR spectra, (**B**) Thermogravimetric analysis, (**C**) UV–visible absorption spectra, (**D**) schematic representation of BNNs coated with P-F127, (**E**,**F**) HRTEM images analysis of the sample BN(T).

The synthesized BNNs were next analyzed by FTIR and TGA. The FTIR spectra of pristine h-BN are relatively similar to those of BNNs produced via tip or bath sonication, as shown in Figure 4A. The non-exfoliated BN as well as BNNs’ spectra present the characteristic peaks at 756 cm^−1^ that are attributed to B−N−B out-of-plane bending vibration and broad absorption bands at 1290 cm^−1^ that is ascribed to the B−N in-plane stretching vibration [12]. These results indicate that the exfoliated BNNs still maintain the hexagonal lattice structure of their starting material, h-BN. Importantly, there was a small shift toward higher wavelengths on these two bands for BNNs when compared to h-BN, which can be attributed primarily to the establishment of interactions during P-F127 intercalation between the BN planes. This is also shown by the bands at 2918 cm^−1^ and 2852 cm^−1^, which are the antisymmetric and symmetric vibrations of the -CH_2_- chains in P-F127, respectively [58]. Additionally, there is the appearance of two small shoulders at 1108 cm^−1^ and 946 cm^−1^ in the case of BNNs, which can be attributed to the stretching vibration of abundant C–O–C in the molecular structure of P-F127 [59]. In the case of BNNs, there is also the presence of a broad band centered at 3404 cm^−1^, which can be attributed to O-H stretching vibrations caused by boron structure oxidation (B-OH) or water molecule sorption [12]. To further investigate the surface functionalization of the exfoliated BNNs, thermogravimetric analysis was performed. The TGA results show that h-BN has a high thermal stability up to 600 °C (Figure 4B). Contrarily, significant weight loss of ca. 4.4% and 6.7% was observed in the cases of BN(B) and BN(T). The superior weight loss of BNNs produced by tip sonication indicates enhanced functionalization with P-F127, which can be attributed to the high degree of exfoliation and consequently a higher specific surface area for the establishment of molecular interactions. According to the literature, graphene oxide functionalized with P-F127 presents a similar initial degradation profile, with a starting point of 200 °C and reaching full degradation at 400 °C [60]. However, it was also reported that P-F127, when intercalated in mesoporous silica particles, can improve thermal stability up to 600 °C [61]. Recently, Chen et al. reported that sucrose presented improved thermal stability when covalently linked to BNNs’ surface [62]. This result allows us to infer that the P-F127 is coating the surface of the BNNs.

Elemental analysis was next performed to further confirm the presence of P-F127 on the samples, and to allow its quantitative determination. BNNs obtained by tip sonication were selected for the analyses as they appeared to be the most promising sample. Table 3 shows the results obtained for the BN(T) in comparison with the as-received h-BN.

As expected, as-received h-BN does not present carbon or hydrogen in its structure, and only nitrogen is detected. The presence of both C and N in the BN(T) sample confirms the coating of the BNNs with P-F127. The C/H ratio is in good agreement with the molecular formula of pluronic acid F127 (see Figure 1) thus confirming that no additional species are present in the sample apart from the BNNs and P-F127. From the C content and taking into account the elemental composition of P-F127, it can be deduced that the sample of BN(T) contains ca. 11 wt. % of P-F127. This value is slightly higher than the determination by TGA but accounts for experimental error of the measurements and batch-to-batch differences in sample preparation.

The UV-Vis absorption spectra of h-BN and BNNs water dispersions at the same concentration (20 µg mL^−1^) presented a very distinct profile (Figure 4C). Non-exfoliated h-BN showed a constant absorption all over the range of wavelengths studied, from 200 up to 1200 nm. In the case of BNNs, a strong absorption peak was clearly identified in the UV region, with a maximum at approximately 205 nm. According to Coleman et al., this peak is characteristic of the π → π∗ transition to BN, and its shift towards higher wavelengths corresponds to the increment in the number of layers. Our findings show that the maximum peak position for BN(B) is 206 nm and for BN(T) is 204 nm. These results confirm the more effective exfoliation using the tip sonication method compared to bath sonication. Additionally, we also observed that the size reduction caused the typical spectral scattering beyond the UV region [63].

The UV-Vis absorption studies of the BNNs in the function of concentration revealed that for higher concentrations there is the appearance of a well-defined absorption shoulder at approximately 325 nm (Figure S4). Defects (bond exciton caused by vacancies) on the BN lattice have previously been shown to induce the formation of shoulders near 220 nm [64]. Based on this previous study, the formation of structural defects on the synthesized BNNs could be excluded because the absorption shoulder appears at higher wavelengths. Another possible cause for the appearance of this shoulder can be attributed to the surface modification of BNNs with P-F127 and the chemical characterization of the resulting nanomaterials. However, the P-F127 had a maximum absorption in water at 220 nm, according to the literature [65]. The presence of other peaks with maximum wavelengths higher than 300 nm in the UV-vis spectrum of BNNs has also been reported. These low-energy lines can be considered optical transitions between van Hove singularities in the one-dimensional density of states of BNNs [66]. The emission spectra of BN(T), for a concentration of 20 μg mL^−1^, in water upon excitation at 300 nm were obtained (Figure S5). The sample exhibited a broad band between 400 and 600 nm and a quantum yield of 1%.

HRTEM of the cross-section of BNNs produced by tip sonication showed that the crystalline structure of BN was preserved during the synthesis process (Figure 4E,F) [62]. The edge regions usually present an amorphous structure order, which can be attributed to a two-fold effect, the tip sonication–induced fracture of BN layers and P-F127 surface functionalization. The central area clearly shows a well-ordered BN lattice structure with an average number of layers of 15 to 20 layers (Figure 4F).

### 3.2. In Vitro Studies

Cytotoxicity studies with HeLa cells were performed to evaluate the effect that the synthesized BNNs have on their viability and internalization. These preliminary data are crucial to determine the potential use of BNNs for biomedical applications. The EZ4U kit was employed as only viable cells are able to reduce tetrazolium salt to formazan ones. Formazan salts present coloration, so the formation of color allows us to determine the cell viability in each case. HeLa cells were incubated with BNNs for 24 h and after the contact time, the EZ4U kit was added and the absorbance of each well was measured. Two different studies were performed to check how the BNNs solutions can affect cell viability. The obtained results showed that BNNs can cause significant interferences with the MTT type studies [43,44].

**Figure 5:**
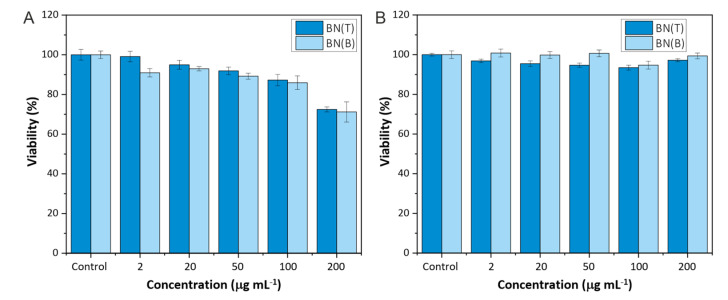
EZ4U assay, (**A**) without removing the medium and (**B**) removing the medium, of HeLa cells incubated with BBNs for 24 h.

Figure 5 shows the results without removing the medium before the addition of tetrazolium salts (A) and removing and adding new medium before the addition of the salts (B). Results show the interference of the synthesized BNNs on the viability values obtained. When the BNNs solution was present during the absorbance measurements, lower viabilities were obtained meanwhile when the medium was changed values close to 100% were obtained in all cases. Therefore, it is important to take into account the possible interference of the BNNs when their toxicity wants to be evaluated by this kind of colorimetric assay. With the obtained results it can be seen that the synthesized nanosheets do not show toxic effects on HeLa cells after 24 h of incubation. More studies have to be performed to further study the interferences as well as to test larger contact times.

Some publications reported the toxic effects of BNNs on different cell lines [14]. In contrast with these previous studies, the exfoliation process followed here to obtain the BNNs coated with P-F127 seems to improve their biocompatibility with HeLa cells after 24 h of incubation.

To further confirm the non-toxicity of the BNNs and again prove their possible interferences on the in vitro studies, flow cytometry was used by staining dead cells with propidium iodide. It is important to take into account again the possible interferences that the material could present. As has been seen with other 2D materials, controls are required when flow cytometry is performed to avoid the misinterpretation of the data [52]. Cytometry results (Figure S6) also show the non-toxic effect of BNNs in HeLa cells after the studied contact time.

**Figure 6:**
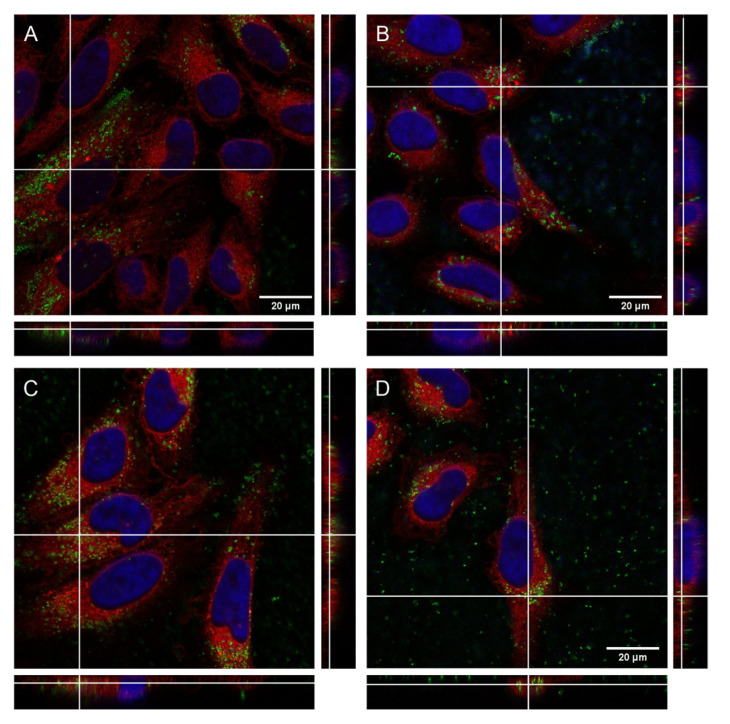
Confocal image with orthogonal projection (white lines) of HeLa cells treated for 24 h with (**A**) BN(B) 50 µg mL^−1^, (**B**) BN(B) 200 µg mL^−1^, (**C**) BN(T) 50 µg mL^−1^ and (**D**) BN(T) 200 µg mL^−1^. BNNs are showed in green, nucleus in blue and cytoplasm in red.

**Figure 7:**
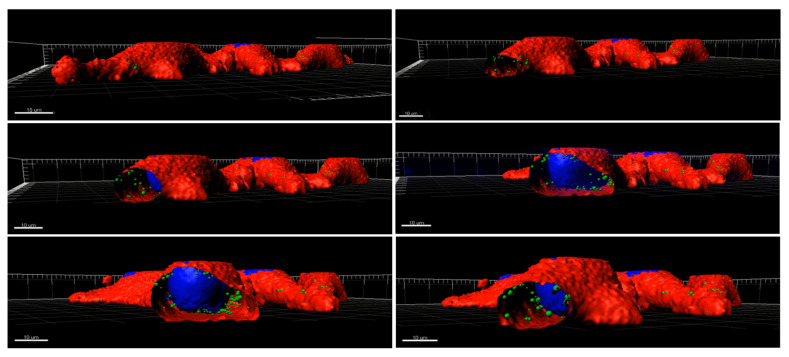
Three-dimensional reconstruction of confocal image of HeLa cells treated with BN(T) 200 µg mL^−1^ for 24 h. BNNs are showed in green, nucleus in blue and cytoplasm in red.

To perform internalization studies, HeLa cells were exposed to 50 µg mL^−1^ and 200 µg mL^−1^ of BNNs for 24 h. Then, cells were fixed and the cell membrane (red) and nucleus (blue) were dyed to allow better interpretation of the BNNs locations inside the cells. Confocal images revealed the presence of the BNNS (in green) inside the HeLa cells’ cytoplasm. Orthogonal projection can be performed to precisely determine the location of nanomaterials in the cells as they allow localizing the compounds in the three dimensions (z, y, z). Figure 6 shows the orthogonal projections, showing a cut on each plane, confirming the uniform distribution of the BNNs inside the cytoplasm of HeLa cells. In addition, a 3D reconstruction of HeLa cells treated with BN(T) 200 µg mL^−1^ for 24 h was performed as can be seen in Figure 7. It clearly confirms the presence of the BNNs inside the cells. The reconstruction movie can be also seen in the supplementary information (Video S1).

**Figure 8:**
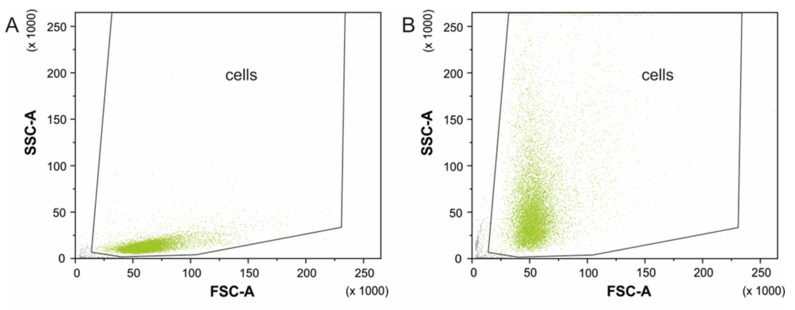
Forward and side scatter profiles for (**A**) HeLa cells without BNNs and (**B**) HeLa cells treated with BN(T) 20 µg mL^−1^ for 24 h. Green dots represents the events defined as cells according to their size (related with FSC) and complexity (related with SSC) and grey dots represents the registered events not defined as cells.

Flow cytometry analysis also allow to confirm the BNNs internalization into the HeLa cells. When nanomaterial is internalized, cell complexity is increased producing an increment on the side scatter signal that is directly related with cell complexity [67,68]. Figure 8 shows dot plot of the cells without BNNs (A) and of the cells treated with BN(T) 20 µg mL^−1^ (B). A high increment on the cell’s complexity can be observed and consequently it can be determined that the nanomaterial have been internalized inside the cells.

## 4. Conclusions

We have presented a novel surface–functionalization-assisted exfoliation of h-BN using P-F127. Few layered BNNs (<10 nm) were obtained with reduced lateral size and narrow size distribution (77.5 ± 1.4 nm). We also observed that the efficiency of the exfoliation process of h-BN is strongly dependent on the presence of the dispersing agent P-F127. At low power (80 W) and adding P-F127 to the reaction medium, it was possible to obtain a similar exfoliation yield (28.0 wt. %) as using a power of 200 W in pure water (30.7 wt. %). The same trend was observed when bath sonication was used; with treatment times of 8 h and in the presence of P-F127, we obtained higher exfoliation yields (27.2 wt. %) than in a 24 h treatment under pure water (25.7 wt. %). These results are in agreement with those of Zhu et al. [21], where it was reported that amphiphilic F68 improves the synthesis of h-BN flakes by promoting both exfoliation by hydrophobic interactions with BNNs’ surfaces and their hydrophilic stabilization in water. Our results showed that using P-F127 with long chains allowed the improvement of the exfoliation yield up to 30 wt. % and obtained stable water dispersions at BNNs’ concentrations of 1.5 mg mL^−1^ for 7 days. The characterization of BNNs revealed that their surface was functionalized with a thin layer of P-F127, which is important for improving water stability and consequently biocompatibility and cell internalization. According to our cytotoxicity studies, HeLa cells had a high viability that was almost 100% at concentrations of 200 µg mL^−1^ of BNNs, prepared by tip or bath sonication. Additionally, we also observed a good cellular internalization of BNNs at different concentrations for 24 h. The high biocompatibility and cell internalization of the synthesized BNNs envision their potential application in the field of boron neutron cancer therapy.

## Figures and Tables

**Table 1:** Conditions for the exfoliation of h-BN by tip and bath sonication. Samples were a large degradation was observed are indicated with “-”.

Method	Experimental Parameters		Yield (wt. %)	
			H_2_O	H_2_O + P-F127
Tip sonication	power	80 W	11.3	28.0
		200 W	30.7	-
Bath sonication	time	8 h	11.2	27.2
		24 h	25.7	-

**Table 2:** Conditions for the exfoliation of h-BN by ball milling.

Experimental Parameters		Yield (wt. %)		
Diameter of the Balls		5.0 mm		1.0 mm
Time		8 h	24 h	8 h
Solvents	Isopropanol (IPA)	13	29	22
	Benzyl benzoate (BB)	9	21	

**Table 3:** Elemental analysis results for the h-BN and BN(T) sample.

Sample	% C	% H	% N
h-BN	<0.1	<0.1	54.68 ± 0.34
BN(T)	6.38 ± 1.21	1.11 ± 0.5	47.99 ± 0.56

## Data Availability

The data presented in this study are available in the article or supplementary material.

## Supplementary Materials

The following supporting information can be downloaded at: https://www.mdpi.com/article/10.3390/biomedicines10123238/s1, Figure S1 and S2: SEM; Figure S3: AFM; Figure S4: Absorbance spectra; Figure S5: Absorption and emission spectra; Figure S6: Flow cytometry; Video S1: In vitro reconstruction movie.
