# Supplementary material for: Sustainable Synthesis of Highly Biocompatible 2D Boron Nitride Nanosheets
Source: Biomedicines. 2022 Dec 13;10(12):3238. doi: 10.3390/biomedicines10123238 (PMC9775030; doi:10.3390/biomedicines10123238)
Supplement: Supplementary file 1 [file biomedicines-10-03238-s001.zip › biomedicines-2070844-supplementary.pdf]

Supporting Information

Large-scale synthesis of highly biocompatible 2D boron nitride nanosheets

1. h-BN exfoliation by ball milling

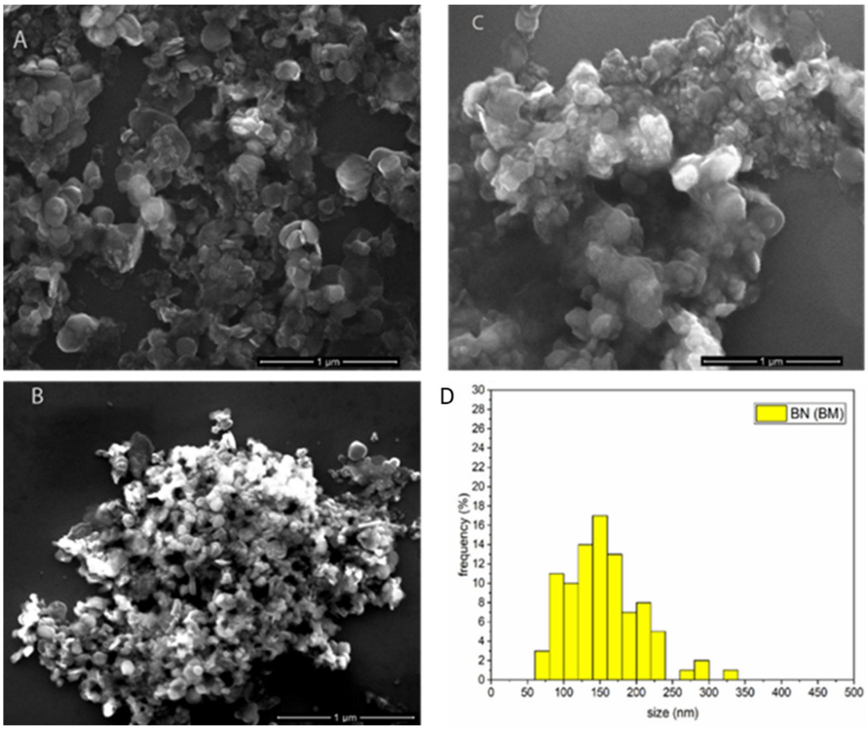

Figure S1.SEM figures of the BNNs obtained by ball milling in IPA: (**A**) 8 h with spheres with 5mm diameter , (**B**) 24h with spheres with 5 mm diameter, (**C**) 8h with spheres with 1 mm diameter . (**D**) Size distribution of BNNs obatined under conditions A.

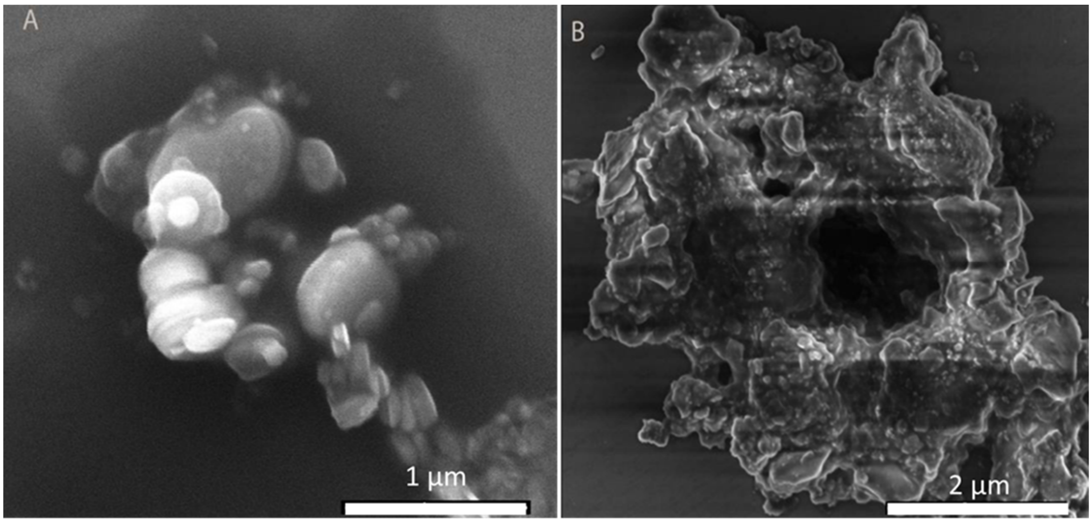

Figure S2. SEM figures of the BNNs obtained by ball milling in BB: (**A**) 8 h with spheres with 5 mm diameter, (**B**) 24h with spheres with 5 mm diameter.

2. Atomic Force analysis

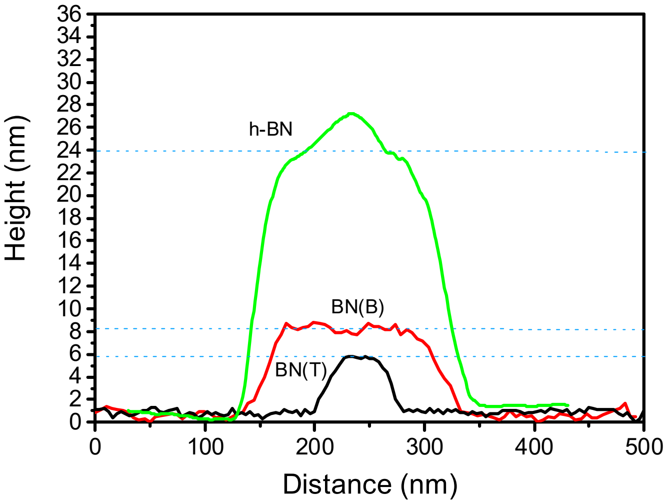

Figure S3. Lateral size, and thickness of the h-BN and exfoliated BNNs from water/Pluronic F127 dispersion by applying tip sonication or bath sonication.

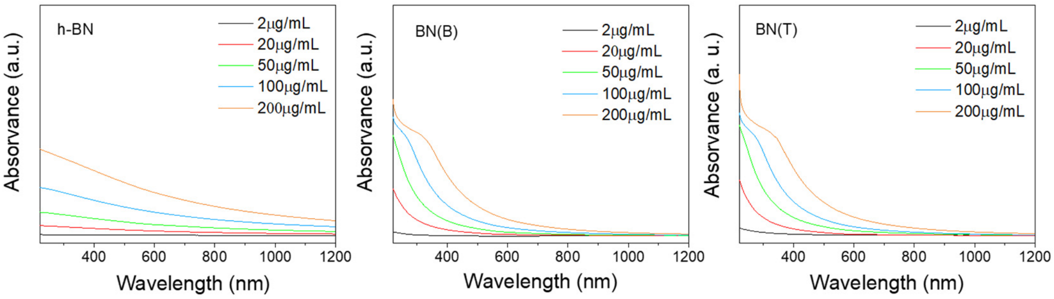

Figure S4. Absorbance spectra of different concentrations of h-BN, BN(B) and BN(T) in water dispersions.

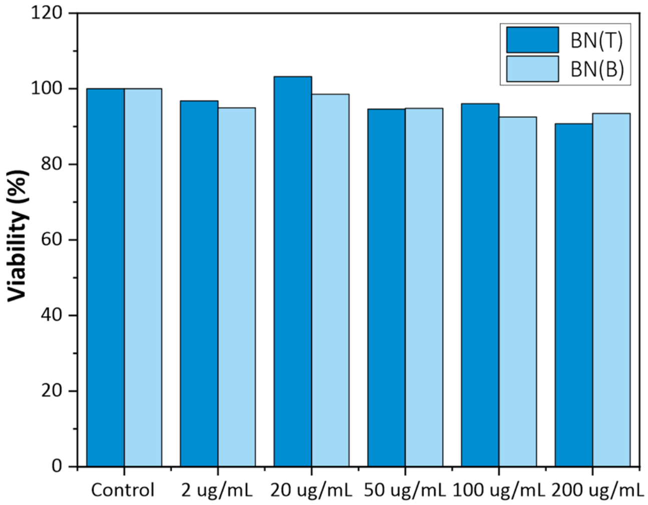

Figure S5. *In vitro* cytotoxicity studied performed with flow cytometry. HeLa cells were incubated with synthetized BN(T) and BN(B) at different concentrations for 24 h and propidium iodide was used to stain dead cells.
